# Meta-Analysis of Studies on the Effects of Digital Therapeutics

**DOI:** 10.3390/jpm14020157

**Published:** 2024-01-30

**Authors:** Young-Chul Seo, Sang Yeol Yong, Won Woo Choi, Sung Hoon Kim

**Affiliations:** 1 Wonju College of Medicine, Yonsei University, 20, Ilsan-ro, Wonju-si 26426, Gangwon-do, Republic of Korea; seoyc08@gmail.com (Y.-C.S.); rehsyyong@yonsei.ac.kr (S.Y.Y.); 2 Department of Rehabilitation Medicine, Yonsei University, 20, Ilsan-ro, Wonju-si 26426, Kangwon-do, Republic of Korea; wwcho465@naver.com

**Keywords:** bias risk assessment, digital therapeutics, effect size, meta-analysis, personalized treatment, treatment outcomes

## Abstract

Digital therapeutics (DTx), novel treatment methods that have the potential to surpass traditional approaches such as pills, have received considerable research attention. Various efforts have been made to explore effective treatment methods that actively integrate DTx. This review investigates DTx treatment outcomes comprehensively through a meta-analysis. The analysis—a manual search of studies on “digital therapeutics”—includes DTx studies from January 2017 to October 2022. Hedges’ g is used to quantify effect size for fifteen studies analyzed, encompassing eight control groups. Further, a quality assessment is performed using the Bias Risk Assessment Tool. The Hedges’ g analysis results provide weighted average effect sizes across the eight control groups, revealing a substantial value of 0.91 (95% CI: 0.62 to 1.20); this signifies a moderate to large effect size. Further refinement, which excludes one study, yields an increased weighted average effect size of 1.13 (95% CI: 0.91 to 1.36). The quality assessment results consistently indicate a low risk of bias across studies. The meta-analysis results indicate that DTx can provide significant pivotal therapeutic impacts and offer a means to personalize treatment approaches and streamline the management of patients’ treatment processes.

## 1. Introduction

Digital therapeutics (DTx) are software-driven therapeutic interventions that combine various types of treatment methods to prevent, control, and treat diseases, ultimately leading to enhanced treatment outcomes [1]. DTx represent an emergent research area within digital healthcare that is distinct from the broader concepts of digital health or digital medicine; DTx extend beyond the realm of conventional small molecular compounds, such as pills and capsules, and biological agents, such as antibodies, proteins, and cells [2,3]. In other words, they utilize “therapeutic intervention” in products. As stated by Sepah et al. [4], behavioral and self-care programs can be considered primary forms of DTx.

Although DTx products have not yet been approved in Republic of Korea, there has been remarkable growth in the global market, and this sector is projected to have an average annual growth rate of 31.4% by 2026 [5]. Regarding DTx products for improving childhood attention deficit hyperactivity disorder (ADHD) symptoms, there have been instances of FDA approval [6] and reports indicating authentic therapeutic effects [7,8]. However, no studies have investigated the average overall therapeutic effect of DTx. While studies on DTx interventions targeting hypertension, body mass index (BMI), and pain, such as those by Jasik [9] and Choi et al. [10], have concluded positive effects, no specific research has been dedicated to examining the overall therapeutic outcomes of DTx.

Therefore, our study is aimed at assessing the overall therapeutic outcomes of DTx use by conducting a meta-analysis, based on data from relevant research fields where DTx has been applied. To achieve this goal, a selection of academic papers was conducted by referencing the studies of Matthews [11], Wu et al. [12], Alipanah et al. [13], and Webb et al. [14]. The overall therapeutic effects reported in each study were investigated based on the provided data. The significance of the therapeutic effects was determined by calculating Hedges’ g, a method first proposed by Hedges in 1981 [15,16,17]. Additionally, a subgroup analysis was performed to discern specific therapeutic effects within each subgroup. Last, a funnel plot was created to assess the homogeneity of the research data and provide an overall evaluation of effect size analysis.

The expected outcomes of our study can be broadly divided into three categories. First, by conducting a meta-analysis based on data from relevant existing studies, we can determine the specific and overall therapeutic effects of DTx. Second, we can establish a framework to assess the actual levels of their efficacy. Third, we can present data that specifically demonstrate the treatment effects on individual symptoms for future research in the field.

## 2. Materials and Methods

### 2.1. Study Selection and Data Extraction

Our study systematically analyzed research findings on the validation of the therapeutic effects attributed to DTx. The analysis focused on studies published between January 2017 and October 2022.

**Figure 1:**
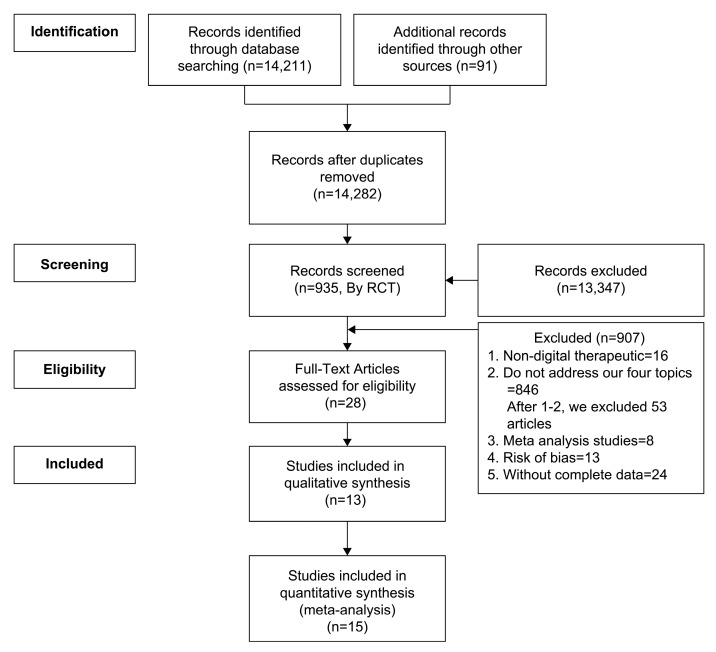
Article selection using the PRISMA protocol.

Adhering to the Preferred Reporting Items for Systematic Reviews and Meta-Analyses (PRISMA) protocol, our literature search involved querying both PubMed and manually identified articles, resulting in 14,211 and 91 articles, respectively (see Figure 1). Additionally, we conducted a manual search of the reference lists from related articles and journals. This search was based on the keywords “digital therapeutics” and “randomized controlled trial”.

To ensure methodological rigor, we employed a thorough cross-validation process between two reviewers. This involved the identification and exclusion of duplicates, papers that did not meet the predefined inclusion criteria, non-DTx research, studies that did not address our topic (substance abuse, musculoskeletal, anxiety, or mental health), any meta-analysis studies, studies with risk of bias, and studies with incomplete data. After considering the research method and data accessibility, we found 13 qualitative studies and a total of 15 articles that were deemed eligible for inclusion in the meta-analysis.

Table 1 presents the list of these 15 selected articles and offers a comprehensive overview of the individual study characteristics. Further, the descriptive statistics summarizing the key aspects of the included studies are provided in Table 2.

The meta-analysis was executed within the framework of the Participants, Intervention, Comparison, Outcome, and Study design (PICOS) model:

- Participants: The focus was on patients necessitating treatment through DTx.
- Intervention: The study encompassed DTx-based experimental interventions.
- Comparison: Comparative data were extracted from experimental groups subjected to DTx.
- Outcome: The primary focus was on assessing the therapeutic effects of DTx.
- Study Design: Studies utilizing randomized controlled trials (RCTs) and pre-post designs were included.

This comprehensive approach allowed for a nuanced examination of the therapeutic landscape of DTx across diverse study designs and participant profiles, providing a robust foundation for the subsequent meta-analysis.

### 2.2. Study Quality Assessment

To determine the risks of bias in each study, the Cochrane Group’s Risk of Bias 2 (ROB2) tool was employed. Each signaling question had the response options of Yes (Y), Probably Yes (PY), Probably No (PN), No (N), and No Information (NI). The assessment categorized bias into three levels: low risk, some concerns, and high risk.

### 2.3. Data Analysis

To analyze the effect sizes, we used Stata 17.0 software. As effect size estimate statistics for group comparisons, standardized mean differences were computed using Hedges’ g with the confidence level set at 95%. Further, we generated a forest plot to evaluate statistical heterogeneity.

## 3. Results

### 3.1. Quality Assessment of the Selected Papers

We conducted a methodological quality assessment on the 15 studies included in the meta-analysis. The evaluation consistently indicated a low risk of bias for 12 of the 15 studies and “some concerns” for 3 of the 15 studies. Consequently, all 15 studies were selected as reliable data sources for further analysis (Table 3).

### 3.2. Meta-Analysis

The investigation into the impact of DTx on patient treatment outcomes, derived from the 15 studies, illuminated significant experimental effects as follows. The overall effects were significant (M = 0.255, 95% CI = 0.000–0.510, *p* < 0.001, Q^2^ = 20.177, I^2^ = 95.044, T^2^ = 0.223), except for those for musculoskeletal and mental health. The heterogeneity among the studies reflects a substantial range (I^2^ = 78.576–96.494). The most prevalent subgroup was anxiety (M = 0.681, 95% CI = 0.194–1.168) and substance abuse (M = 0.591, 95% CI = 0.133–1.049). Furthermore, an Egger’s test showed that there were no significant biases in the included studies (*p* = 0.095).

Table 4 presents the results of investigations into the effects on various subgroups and diseases, and the combined results of selected studies categorized by subgroups, which provide insights into the pooled effects across different domains, are presented in Table 5.

## 4. Discussion and Conclusions

The current meta-analysis systematically evaluated the therapeutic effects of DTx by rigorously analyzing 15 independent studies. Employing stringent screening processes and carefully addressing issues related to heterogeneity, our findings unequivocally demonstrated the significant positive impacts of DTx on patients’ overall health outcomes. Specifically, the heterogeneity between subgroups can be useful for comprehensively examining the effects of DTx, including the evaluation and comparison of DTx between subgroups. Moreover, the related results can be used to encourage DTx-related research activities in each subgroup and further expand related technologies. The analysis demonstrated that DTx exhibited a remarkable reduction in adverse effects, especially in alleviating pain (effect size = 0.255, *p* = 0.049, 95% CI = 0.000–0.389). This finding holds significant implications for improving patients’ quality of life and reducing healthcare costs associated with pain management.

A further breakdown by disease group revealed intriguing insights. We observed substantial therapeutic effects in studies on substance use disorders [21] and anxiety associated with peripheral intravenous cannulation [22]. The analysis of effect sizes indicated significant enhancements in perception measures (FPS-P, GAD-7, and HADS). These findings underscore the potential of DTx as valuable treatment methods for addressing substance abuse and anxiety-related disorders.

Notably, the five studies focusing on musculoskeletal disorders did not yield statistically significant effects; similarly, the four studies centered on mental health did not exhibit substantial impacts either. The lack of effectiveness of DTx in such disease categories may relate to the limitations of treatment methods through behavioral interventions versus those that act directly on the body. This may be particularly true for diseases and techniques that do not fully reflect individual and subjective physical and mental characteristics.

Specifically, in mental health-related studies, the intricate interplay of individual psychological states with various contextual elements may contribute to the observed lack of significant effects. Mental well-being is inherently subjective and influenced by diverse personal experiences and environments. Furthermore, if DTx do not present comprehensive solutions for specific mental health conditions, alternative or complementary approaches may be warranted. A holistic approach that integrates DTx with traditional therapeutic methods could yield more promising outcomes for certain mental health conditions. Regarding musculoskeletal disorders, it is possible that DTx may not provide the requisite level of physical intervention necessary for this particular condition. These conditions often necessitate tailored physical therapeutics, exercises, and other hands-on treatments, which DTx may not adequately incorporate them. A combination of DTx and traditional physical therapeutic approaches could potentially address the limitations of either method.

However, DTx can be considered as effective approaches for the management of substance abuse and anxiety, particularly when implemented through well-structured behavioral programs and self-care in specific fields. Our findings underscore that DTx interventions have significant positive health outcomes, further highlighting their therapeutic feasibility. Additionally, the meticulous application of the PRISMA protocol here helped us identify future directions for presenting DTx-related research findings. Our study emphasized the importance of precise result presentation and the establishment of universally acceptable criteria to further validate and demonstrate the effects of DTx. Further, it should be noted that the meta-analysis conducted in this study provided valuable insights into the overall research trends and outcomes regarding the therapeutic effects of DTx.

However, our study does have some limitations. First, the diversity of the various research designs and measurement tools makes consistent analyses and interpretations difficult. Second, securing sufficient reliability was challenging, based on the limited number of studies in a specific disease category. That is, our analyses may be influenced by various study characteristics, designs, and factors, such as sample size, study duration, types of DTx employed, and measurement tools. It is therefore crucial to acknowledge that the results may vary depending on the target population and disease categories. To ensure the generalizability of the study findings, it is imperative to track ongoing research and validate the results over time. To further enhance our understanding of DTx, it is necessary to diversify research areas and explore their strengths and weaknesses in various fields. This comprehensive approach will enable the identification of effective treatment methods and strategies tailored to specific conditions and patient needs. Moreover, it is necessary to standardize study designs and methodologies to minimize the influence of these confounding factors and provide more definitive conclusions regarding the efficacy of DTx across different disease categories.

DTx technology and related markets are emerging rapidly with the concurrent development of science and technology. Their potential for improving the quality of life of numerous patients is anticipated to grow exponentially in the future, along with the efficient employment of advanced technologies. However, at the same time, there may be several barriers to adopting and applying DTx. These include high development costs, technical difficulties, traditional resistance from the medical community, and the attitudes of patients and medical personnel. To overcome these barriers, integrated cooperation is required in various aspects, such as the effective integration with the existing medical system, the improved understanding of medical personnel through education and training programs, and the introduction of cost-effective models.

## Figures and Tables

**Table 1:** General information on selected studies.

Study	System Used	Subgroup	Follow-Up Duration	Target	Indicators	N (Control/ Post)	N (Experiment/Post)
Prochaska et al. [18]	Woebot-SUDs (W-SUDs)	Substance abuse	30 days	Screened positive for substance misuse (CAGE-AID > 1)	GAD-7	71 (−1.6 ± 0.6)	81 (−0.70 ± 0.50)
Kor and Shoshani [19]	School-based Positive Psychology Addiction Prevention (PPAP)	Substance abuse	12 months	School children	GSI	833 (15.06 ± 12.57)	837 (21.04 ± 15.71)
Ahlers et al. [20]	CANreduce 2.0	Substance abuse	6 weeks	Cannabis use at least once weekly over the last 30 days (adults)	GAD-7	94 (4.71 ± 3.53)	273 (6.37 ± 3.56)
Mujcic et al. [21]	MyCourse, a digital AM (alcohol moderation) intervention	Substance abuse	At 3, 6, 9, and 12 months	Adult 10-year cancer survivors drinking over the Dutch-recommended drinking guidelines (≤7 standard units (10 g of alcohol) per week) with the intention to moderate or quit drinking	AUDIT	53 (10.0 ± 6.0)	50 (9.30 ± 5.10)
Nelligan et al. [22]	Custom-built website with information on OA	Musculoskeletal	24 weeks	Clinical criteria for knee OA in communities across Australia from July 2018 to August 2019	AQoL-6D	103 (0.75 ± 0.16)	103 (0.69 ± 0.20)
Tore et al. [23]	Video conference	Musculoskeletal	8 weeks	Diagnosed with moderate/mild KOA	HADS	24 (7.00 ± 4.07)	24 (12.33 ± 5.66)
Weise et al. [24]	Digital home exercise program	Musculoskeletal	12 weeks	Participants with unspecific and degenerative back pain aged ≥ 18 years	German VNRS	105 (−0.91 ± 1.50)	108 (−3.35 ± 2.05)
Gold et al. [25]	VR intervention	Anxiety	3 months	Patients aged 10 to 21 years who were undergoing PIVC placement	FPS-P	53 (1.09 ± 1.82)	54 (2.19 ± 2.21)
Roy et al. [26]	The app-delivered MT program	Anxiety	2 months	Using social media advertisements	GAD-7	28 (4.8 ± 4.1)	33 (10.60 ± 3.50)
Shaffer et al. [27]	Behavioral therapy for insomnia (CBT-I)	Anxiety	Up to 12 months	Adults aged ≥ 55 with insomnia	HADS	192 (5.26 ± 3.56)	97 (6.42 ± 3.60)
Kannampallil et al. [28]	Virtual voice-based coach	Anxiety	16 weeks	Adults with mild-to-moderate depression and/or anxiety	HADS	40 (−2.52 ± 4.48)	21 (0.14 ± 5.11)
Espie et al. [29]	Digital cognitive behavioral therapy (dCBT)	Mental Health	24 weeks	Just recruited	GAD-7	853 (4.70 ± 4.21)	858 (6.05 ± 4.50)
Araya et al. [30]	CONEMO (English translation, emotional control)	Mental Health	3 months (Sao Paulo)	Being treated for hypertension and/or diabetes	EQ-5D	389 (0.68 ± 0.19)	396 (0.65 ± 0.19)
Han et al. [31]	Conference software, Tencent	Mental Health	3 months	LGBTQ+ young adults aged between 18 and 29 who scored moderate or above on at least one dimension of the Depression Anxiety Stress Scale 21 and did not have help-seeking experiences in the past 12 months	GHSQ	68 (2.95 ± 0.50)	69 (2.77 ± 0.56)
Comtois et al. [32]	App-based	Mental Health	4 weeks	Unemployed because of COVID-19, or were COVID-19-designated essential workers	GAD-7	151 (7.80 ± 5.60)	151 (7.30 ± 5.80)

GAD-7 (Generalized Anxiety Disorder-7); GSI (Gray Scale Inversion); AUDIT (Alcohol Use Disorder Identification Test); AQoL-6D (Assessment of Quality of Life-6D); HADS (Hospital Anxiety and Depression Scale); German VNRS (Verbal Numerical Rating Scale); FPS-P (Facial Pain Scale); EQ-5D (Euroqol 5 Dimensions); GHSQ (General Help-Seeking Questionnaire).

**Table 2:** Descriptive statistics of selected studies.

Category	Control	Experiment
N	204	210
Mean	4.00	5.45
Standard Deviation	3.52	3.83

**Table 3:** Quality assessment of the selected papers.

Author and Year	1.1 Was the Allocation Sequence Random?	1.2 Was the Allocation Sequence Concealed Until Participants Were Enrolled and Assigned to Interventions?	1.3 Did Baseline Differences between Intervention Groups Suggest a Problem with the Randomization Process?	Risk of Biased Judgment
Prochaska et al. [18]	PN	Y	Y	Some concerns
Kor and Shoshani [19]	Y	Y	N	Low Risk
Ahlers et al. [20]	Y	Y	N	Low Risk
Mujcic et al. [21]	Y	Y	Y	Low Risk
Nelligan et al. [22]	Y	Y	Y	Low Risk
Tore et al. [23]	Y	Y	Y	Low Risk
Weise et al. [24]	Y	Y	Y	Low Risk
Gold et al. [25]	Y	Y	Y	Low Risk
Roy et al. [26]	Y	Y	Y	Low Risk
Shaffer et al. [27]	Y	Y	Y	Low Risk
Kannampallil et al. [28]	Y	Y	N	Some concerns
Espie et al. [29]	Y	Y	Y	Low Risk
Araya et al. [30]	PN	Y	Y	Some concerns
Han et al. [31]	Y	Y	Y	Low Risk
Comtois et al. [32]	Y	Y	Y	Low Risk

**Table 4:** Meta-analysis of the selected studies.

Studies	Subgroup	d_s_	95% CI		Weight (%)	*p*
			LL	UL		
Ahlers et al. [20]	Substance abuse	0.466	0.229	0.703	7.14	<0.001
Mujcic A et al. [21]	Substance abuse	−0.125	−0.511	0.263	6.41	0.526
Nelligan RK et al. [22]	Musculoskeletal	−0.330	−0.605	−0.055	6.98	0.018
Tore NG et al. [23]	Musculoskeletal	1.064	0.453	1.664	5.17	<0.001
Gold JI et al. [25]	Anxiety	0.539	0.152	0.924	6.41	0.006
Shaffer KM et al. [27]	Anxiety	0.324	0.078	0.569	7.11	0.01
Kannampallil T et al. [28]	Anxiety	0.502	−0.036	1.036	5.56	0.064
Espie CA et al. [29]	Mental health	0.310	0.214	0.405	7.58	<0 .001
Araya R et al. [30]	Mental health	−0.158	−0.298	−0.018	7.48	0.027
Han M et al. [31]	Mental health	−0.337	−0.674	0.001	6.67	0.049
Roy A et al. [26]	Mental health	−0.088	−0.313	0.138	7.19	0.447
Comtois KA et al. [32]	Anxiety	1.512	0.935	2.080	5.35	<0 .001
Prochaska et al. [18]	Substance abuse	1.631	1.261	1.997	6.51	<0.001
Kor and Shoshani [19]	Substance abuse	0.420	0.323	0.517	7.58	<0.001
Weise et al. [24]	Musculoskeletal	−1.351	−1.647	−1.052	6.87	<0 .001

Notes: Explanation of abbreviations and symbols: Studies: Number of studies; Subgroup: Subgroup classification; ds: Effect size; 95% CI: 95% Confidence Interval; Weight (%): Weight percentage; *p*: Significance level; LL: Lower limit of the confidence interval; UL: Upper limit of the confidence interval.

**Table 5:** Meta-analysis of the selected studies by subgroup.

Subgroups	Studies (df)	Effect Size	95% CI		*p*	Q^2^	Q^2^-*p*	I^2^	T^2^
			LL	UL					
Substance Abuse	4 (3)	0.591	0.133	1.049	0.011	16.100	<0.001	93.789	0.221
Musculoskeletal	3 (2)	−0.320	−0.873	0.232	0.256	28.518	<0.001	96.494	0.904
Anxiety	4 (3)	0.681	0.194	1.168	0.006	4.668	0.003	78.576	0.156
Mental Health	4 (3)	−0.059	−0.507	0.389	0.796	13.519	<0.001	92.603	0.089
Overall	15 (14)	0.255	0.000	0.389	0.049	20.176	<0.001	95.044	0.221

Notes: see Table 4.

## Data Availability

No new data were created or analyzed in this study. Data sharing is not applicable to this article.
